# Modeling orientation perception adaptation to altered gravity environments with memory of past sensorimotor states

**DOI:** 10.3389/fncir.2023.1190582

**Published:** 2023-07-20

**Authors:** Aaron R. Allred, Victoria G. Kravets, Nisar Ahmed, Torin K. Clark

**Affiliations:** ^1^Bioastronautics Laboratory, Smead Department of Aerospace Engineering Sciences, University of Colorado Boulder, Boulder, CO, United States; ^2^Cooperative Human-Robot Interaction Laboratory, Smead Department of Aerospace Engineering Sciences, University of Colorado Boulder, Boulder, CO, United States

**Keywords:** vestibular, otolith, multisensory integration (MSI), internal model (IM), Bayesian, astronaut

## Abstract

Transitioning between gravitational environments results in a central reinterpretation of sensory information, producing an adapted sensorimotor state suitable for motor actions and perceptions in the new environment. Critically, this central adaptation is not instantaneous, and complete adaptation may require weeks of prolonged exposure to novel environments. To mitigate risks associated with the lagging time course of adaptation (e.g., spatial orientation misperceptions, alterations in locomotor and postural control, and motion sickness), it is critical that we better understand sensorimotor states during adaptation. Recently, efforts have emerged to model human perception of orientation and self-motion during sensorimotor adaptation to new gravity stimuli. While these nascent computational frameworks are well suited for modeling exposure to novel gravitational stimuli, they have yet to distinguish how the central nervous system (CNS) reinterprets sensory information from familiar environmental stimuli (i.e., readaptation). Here, we present a theoretical framework and resulting computational model of vestibular adaptation to gravity transitions which captures the role of implicit memory. This advancement enables faster readaptation to familiar gravitational stimuli, which has been observed in repeat flyers, by considering vestibular signals dependent on the new gravity environment, through Bayesian inference. The evolution and weighting of hypotheses considered by the CNS is modeled via a Rao-Blackwellized particle filter algorithm. Sensorimotor adaptation learning is facilitated by retaining a memory of past harmonious states, represented by a conditional state transition probability density function, which allows the model to consider previously experienced gravity levels (while also dynamically learning new states) when formulating new alternative hypotheses of gravity. In order to demonstrate our theoretical framework and motivate future experiments, we perform a variety of simulations. These simulations demonstrate the effectiveness of this model and its potential to advance our understanding of transitory states during which central reinterpretation occurs, ultimately mitigating the risks associated with the lagging time course of adaptation to gravitational environments.

## 1. Introduction

In humans, exposure to a new gravity environment results in a central reinterpretation of information from multiple sensory sources, producing an adapted sensorimotor state appropriate for motor actions and spatial orientation perceptions in the new environment (Clark, 2019). However, the temporal evolution of this central adaptation is not instantaneous, and it can take weeks of prolonged exposure to novel environments for adaptation to complete (Roll et al., 1993; Mulavara et al., 2010; Wood et al., 2015).

In the case of astronauts newly exposed to microgravity, the lagging time course of adaptation results in perceptual and functional deficits, including spatial orientation misperceptions and alterations in locomotor and postural control (Clément and Reschke, 2008). This sensorimotor impairment can impact crewmembers’ ability to perform mission-critical operational tasks such as piloting vehicles and operating other complex systems (Paloski et al., 2008). Further, it is thought that space motion sickness (SMS) is largely driven by an adapting central nervous system (CNS) incorrectly expecting self-orientation sensory information for vestibular signals of self-motion (Oman, 1982; Lackner and DiZio, 2006). Concerning SMS, symptoms often exceed mere discomfort (Oman, 1987; Davis et al., 1988; Heer and Paloski, 2006), and the risk of nausea and emesis dictates operational schedules and extravehicular activity timelines (Jennings, 1998). Further, sensorimotor adaptation to changing gravitational stimuli also occurs when exiting the microgravity environment [e.g., transitioning to the Earth, Lunar (Clark, 2022), and Martian surfaces], leaving crewmembers once again maladapted and presenting an expected hinderance to future space exploration missions.

To mitigate these risks, it is critical that we better understand transitory central states during which central reinterpretation occurs. With only a conceptual understanding of this adaptation process, we cannot make the operational decisions (e.g., timing of extravehicular activities) necessary to ensure the safety and performance of the crew. To this end, a computational model of human perceptions of self-motion emulating sensorimotor adaptation to new gravity stimuli is needed. Such a model of the CNS enables evaluating perceptual changes, assessing operational risks, and ultimately implementing appropriate countermeasures. Recently we developed a computational model of the neural mechanisms that may be necessary to adapt to altered gravity environment. As detailed below, this approach used fixed parallel alternative hypotheses for the magnitude of gravity, the resulting sensory conflict for each, and Bayesian updates to drive adaptation but did not include a means to develop, update, or retain alternative hypotheses for the magnitude of gravity (Kravets et al., 2021). As an initial step to address this limitation, we have since enhanced the modeling framework to include the ability to dynamically learn new hypotheses of gravity (Kravets et al., 2022). However, this initial implementation was naïve to any prior internal estimate history. The work presented here extends the computational means by which the CNS may dynamically learn and consider alternative hypotheses of gravity by modeling learned states consolidated into implicit memory from prior adaptations.

### 1.1. Background

Our perception of orientation in three-dimensional space is the result of the complex interaction between our body’s real-world dynamics and our central estimation of these dynamics. Our inertial orientation and self-motion are primarily sensed by noisy sensors in the inner ear (i.e., semi-circular canals and otolith organs), which generate sensory afference. To make sense of these noisy, sometimes ambiguous measurements, our brain relies on internal models of sensory dynamics that generate expected afference (Merfeld et al., 1993, 1999; Merfeld and Zupan, 2002; Tin and Poon, 2005). When there are disparities between actual and expected afference, vestibular “sensory conflict” arises, which is thought to drive dynamic updates of the states in the internal model (Oman, 1982; Oman and Cullen, 2014). Studies by Roy and Cullen (2004), Brooks and Cullen (2009), and Jamali et al. (2009) have identified neurons which differentially respond to passive vs. active (i.e., where the brain can generate appropriate expected afference) self-motion (specifically the behavior of “Vestibular Only” neurons) in the vestibular nuclei and cerebellum. These responses are analogous to the hypothesized sensory conflict signal within the observer framework. Apart from the vestibular sense, sensory conflict is also thought to reside in other sensory systems [e.g., “residual error” signals found in the visual cortex within the “predictive coding” framework (Srinivasan et al., 1982; Rao and Ballard, 1999; Huang and Rao, 2011)].

To formalize and quantitatively describe these theories, computational models such as the “observer” model of spatial orientation perception have been developed (Merfeld et al., 1993; Merfeld and Zupan, 2002; Karmali and Merfeld, 2012; Clark et al., 2019). Observer uses sensory conflict signals to drive central estimates of orientation perception by comparing noisy sensory measurements to expected afference signals, based on internal model computations (Brooks et al., 2015; Carriot et al., 2015). Observer has been experimentally shown to predict self-orientation and motion perceptions in a variety of Earth 1 g (Merfeld et al., 1993; Haslwanter et al., 2000; Zupan et al., 2000; Newman, 2009), hyper-gravity (Clark et al., 2015b,c), and hypo-gravity (Clark and Young, 2017; Galvan-Garza et al., 2018) motion paradigms. However, the observer model does not treat the magnitude of gravity as a dynamic parameter but instead as a fixed one. In general, these model parameters represent neural circuitry of spatial orientation perception in a static 1 g environment where adaptation is not necessary.

However, if the environment changes (e.g., hyper-gravity), within the framework of the model, one might expect the model’s parameters to evolve. Following a gravity transition a gravity transition, sensory information is altered, rendering existing internal models inappropriate: primarily, the models used to disambiguate forces due to linear acceleration and gravity within the gravito-inertial force (GIF) vector which is sensed by the otolith organs (since the vestibular system, by Einstein’s equivalence principle, cannot directly sense gravity). The sensory conflict resulting from inappropriate internal models (for the new environment) is thought to drive a dynamic reinterpretation of vestibular signals, or “adaptation” of the internal models, appropriate for the new gravity environment. Because we still have a limited understanding of the neural computations involved in this process, modeling this neurovestibular adaptation to altered gravity continues to be an active area of scientific interest.

#### 1.1.1. Existing models

Since the late 1990s, computational models of humans adapting to changing gravity stimuli have emerged. A series of works found human disambiguation of the sensed GIF to likely be achieved through internal models that help track the gravity vector’s direction (for a static magnitude of gravity) using cues from the semicircular canals (Merfeld et al., 1999, 2001; Zupan et al., 2000; Merfeld and Zupan, 2002); this theory is referred to as the GIF resolution hypothesis. The implementation of this theory, however, does not enable the CNS to update the magnitude of gravity (or other learned parameter model constants e.g., model gains and time constants). Another prominent hypothesis utilized to model this disambiguation is the frequency segregation hypothesis; Bos and Bles (1998) proposed the subjective vertical conflict (SVC) model which utilizes a low-pass filter to estimate both the magnitude and direction of gravity by the CNS in conjunction with Mayne’s (1974) principle to track the head’s position in an exocentric coordinate system. While the SVC model provides the additional flexibility of a dynamically estimated magnitude of gravity [compared to models built on the GIF resolution hypothesis (Groen et al., 2022), such as the observer model, which nominally assume a constant internal magnitude of gravity], the internally estimated magnitude of gravity adapts in approximately tens of seconds [which is likely unrepresentative of the central reinterpretations involved during altered gravity adaptation, which spans hours to days (Clark, 2019)]. Furthermore, the SVC model offers no framework updating learned parameters or modeling memory of previously experienced environments. While a fundamentally different modeling effort, we note that Wada (2021) aims to capture how the brain might learn exogenous motion dynamics, modifying the expected sensory feedback.

To circumvent these issues, it has been hypothesized that the CNS relies on Bayesian inference to update internal model parameters (Körding and Wolpert, 2004; Darlington et al., 2018), a theory that broadly explains how the CNS uses evidence to reorganize synaptic connections (e.g., reinterpret sensory signals after acute damage to vestibular sensors or downstream afferent nerves, update forward models (i.e., motor memory) after efferent central nervous damage, etc.). Applying this hypothesis to how the CNS may update the magnitude of gravity, an internal model parameter that is rarely stimulated on Earth, multiple iterations of Bayesian models have emerged: first a Bayesian-based computational framework for explaining how the CNS can utilize sensory conflict from the observer model to achieve this update (Kravets et al., 2021), and later a particle filter implementation of this framework to explain how the CNS can achieve this update while considering a finite, dynamic set of alternative hypotheses (Kravets et al., 2022).

While these models of vestibular adaptation to changing gravitational stimuli are well suited for exposure to novel stimuli not-yet experienced by the individual, they have yet to distinguish how the CNS reinterprets sensory information from familiar environmental stimuli (i.e., readaptation). As detailed in the next section, spanning both aerospace and terrestrial applications, there exists evidence that readaptation back to a “learned state,” acquired through long-term memory (LTM), enables more rapid readaptation in humans.

#### 1.1.2. Evidence of learned states for readaptation and neuroplasticity

Historically for shuttle astronauts transitioning from microgravity to Earth gravity, postural performance, signaling readaptation to Earth gravity, has been found to be better in repeat flyers than first-time flyers (Reschke et al., 1998; Paloski et al., 1999) suggesting that exposure to familiar gravitational stimuli results in faster adaptation rates. Because Paloski et al. (1999) found significantly better performance in repeated flyers compared to first-time flyers post-flight (with no differences in pre-flight performance) on the sensory organization balance tests 5 and 6 (with no differences on tests 1–4), they suggest that repeat flyers may be “dual adapted and able to more readily transition from one set of internal models to the other.” Additionally, deconditioning of the neural circuitry comprising the vestibulo-ocular reflex pathway (measured via ocular counter-roll) shortly after transitioning from microgravity to Earth gravity (1–3 days) has been found to be negatively associated with the number of prior flights in crewmembers (Schoenmaekers et al., 2022). This finding may also be indicative of more flight experience resulting in faster readaptation, and Schoenmaekers et al. hypothesize that experienced flyers acquire a central adaptation from previous space flight missions. Supporting this idea, Gonshor and Jones (1976) found more rapid readaptation of this reflex following a prolonged (2–4 weeks) adaptation to a novel vision reversal stimulus.

Together, these findings support the conceptual idea that the CNS relies on learned states (i.e., a memory of internal circuitry / parameters) to achieve faster readaptation to a familiar gravitational stimulus. However, this evidence could be alternatively interpreted as the CNS becoming more adept at searching for new states (i.e., exhibiting faster adaptation to all stimuli, novel or familiar- that is, learning-to-learn) with repeated adaptations. Further supporting the notion of learned states, long-term postural control adaptation (with consolidated motor strategies) in the presence of artificial perturbations has been found to be isolated to the specific test conditions facilitating adaptation (Tjernström et al., 2002). Regarding adaptation to multiple novel altered-gravity environments, there is evidence that recent exposure to one novel gravity (1.5 or 2 g) results in more rapid adaptive adjustments to baseline performance in a second novel gravity (2 or 1.5 g, respectively) (Clark et al., 2015a). However, Clark et al. (2015a) hypothesizes that this effect is likely cognitive/strategic vs. a restructuring of neural circuitry due to the relatively short time course of adaptation.

Despite this evidence of sensorimotor learning and memory, we do not currently know the neural mechanisms by which parameters involved with learned environments are stored. In the more specific case of motor learning, short-term motor memory is thought to arise in cerebellar Purkinje neurons, and through the consolidation process, short-term adapted strategies are eventually stored as long-term motor programs that can be recalled via neural circuits in the vestibular nuclei [in the case of the horizontal optokinetic response (Shutoh et al., 2006)] and basal ganglia [in the case of stimulus-response associations and motor habits (Packard and Knowlton, 2002)]. However, long-term storage of parameters in the self-orientation perception model, such as a magnitude of gravity, may occur elsewhere, likely distributed across brain regions (i.e., the multiple memory systems theory). For instance, cortical changes associated with central adaptation in F-16 pilots (Radstake et al., 2023), who are repeatedly exposed to altered gravity environments, may provide additional insight.

### 1.2. Objectives

Based on these findings, we aim to enhance existing models by providing multiple theoretical additions to existing works. These additions provide phenomenological mechanisms the CNS can utilize to achieve faster sensorimotor readaptation through the consideration of learned states. We aim to model these mechanisms by building on the recent work of Kravets et al. (2022), an implementation of the COMPASS framework (Kravets et al., 2021) where the CNS does not consider a static set of potential gravity magnitude hypotheses. Instead, the evolution of hypotheses considered by the CNS is modeled via an indirect sampling approach, employing a Rao-Blackwellized particle filter algorithm.

## 2. Proposed theory

Bolstering the findings of others in our field, we propose that the CNS is capable of consolidating internal model information describing the current gravitational environment into long-term memory, provided the state is harmonious (i.e., produces low levels of vestibular sensory conflict). Following this storage, the CNS then leverages information about these past states to more readily readapt in the presence of previously experienced stimuli. Using only vestibular sensory conflict, without additional sensory modality (e.g., vision, somatosensory) or ground truth information, we build our theory on the fundamental idea that the CNS considers alternative hypotheses and weighs their likelihoods to formulate an estimate (i.e., uses Bayesian inference) for updating internal models. We give form to this proposed theory through the model framework presented in this section.

In summary, this framework provides a new form of the hypothesis sample distribution (set to be the state transition probability) that is influenced by prior learned internal state parameters. This addition enables faster readaptation to familiar environmental stimuli. Secondarily, we include a formulation for the temporal evolution of learned internal state parameters, enabling the emergence, prioritization, and cessation of learned states within the CNS.

### 2.1. Model framework overview

Dynamic state estimation during gravity adaptation can be modeled using a particle filter, which uses Monte Carlo methods to estimate a state space over time. In contrast to a broad, static (pre-defined) set of alternative hypotheses, a particle filter relies on a smaller, dynamic set that spans different regions of the state space over time. Such an approach provides the CNS a more computationally efficient means to generate an estimate (no longer having to consider unlikely hypotheses) and additionally enables considering new hypotheses outside the domain of what has previously been considered. However, any sensory learning process will involve a complex, multidimensional state space, and estimates driven entirely by Monte-Carlo simulations are likely still too computationally expensive and inefficient to be utilized for the entire state space during such scenarios. Critically, “vanilla” particle filter algorithms (i.e., where sampling of all states is just performed in a Monte-Carlo fashion) fail to leverage the well-validated observer model, in which perceived states (e.g., angular velocity, gravity, linear acceleration) are produced via sensory conflict and internal models. Alternatively, a Rao-Blackwellized Particle filter (RBPF) makes use of these known relationships, analytically estimating “easy” state variables and conditioning those computations on Monte Carlo estimates of “hard” variables (Ristic et al., 2003; Doucet et al., 2013). We propose that the CNS relies on a similar partitioning approach of the state space.

Using the RBPF framework, we represent the CNS adaptation to altered gravity through indirect sampling of potential alternative hypotheses for the magnitude of gravity ($\left|\vec{g}\right|$, a “hard” variable, a model parameter representing neural circuitry), while estimating the remaining “easy” state variables (e.g., the perception of angular velocity, linear motion, and the direction of gravity). The full estimation of self-orientation perception as a posterior probability distribution is conditionally factorized as the following:


$$p\,\left({\text{X}}_{k}|Y_{1:k}\right)=p\,\left(x_{k}|Y_{1:k}\right)\cdot p\,\left({\text{X}}_{k}\backslash \left\{x_{k}\right\}|x_{k},Y_{1:k}\right)$$

Here, ***X_k_*** is the full state space vector at time step *k*, and in the case of vestibular-driven gravity adaptation, the observer framework is used to estimate the *x*, *y*, and *z* components of linear acceleration and angular velocity, ***X_k_***\{*x*_*k*_} (the full set of state parameters excluding the internal estimate of the magnitude of gravity), and each observer solution is conditioned on the sampled parameter, *x*_*k*_, the internal hypothesis of the magnitude of gravity. *Y*_*1:k*_is the complete set of sensory measurements (e.g., from the otoliths and semicircular canals) up to time step *k* (*Y*_1:*k*_ = {*y*_1_, …, *y*_*k*_}).

**FIGURE 1:**
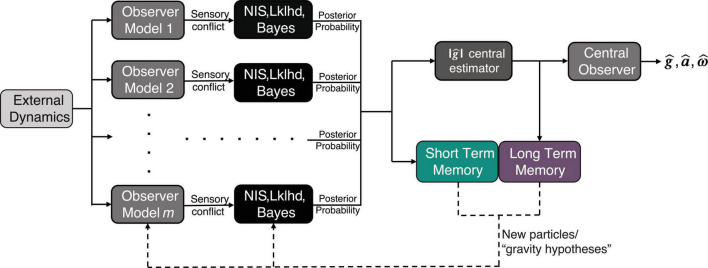
Model Framework for adaptation to altered gravity incorporating short term memory (STM) and long-term memory (LTM). The inputs to the model are a time history of body/world dynamics [x, y, and z components of linear acceleration (***a***) and angular velocity (*ω*)], and the outputs are the perceived gravity $\left(\hat{\text{g}}\right)$, acceleration $\left(\hat{\text{a}}\right)$, and angular velocity $\left(\hat{\omega }\right)$. Here and throughout the text, bold denotes a 3D vector.

Conceptually, the algorithm can be visualized by Figure 1. As proposed in Kravets et al. (2021), at each time step, the algorithm considers multiple hypotheses for the magnitude of gravity, or “particles,” in parallel, and conditions an observer model on the internal estimate from each of these hypotheses. Using its hypothesis of the magnitude of gravity, each observer generates expected afferent signals (i.e., for the semicircular canals and otoliths). Each expected afferent signal is compared to the actual afferent measurement (which is the same for each of the parallel observers), and the difference between the two (actual and expected) is captured in a set of multidimensional sensory conflict signals [***e***_*a*_, ***e***_*f*_, ***e***_ω_, using the standard convention for conflicts associated with linear acceleration, GIF, and angular velocity, see Clark et al. (2019)].

The sensory conflict signals from each observer are combined into a unidimensional Normalized Innovation Squared (NIS) Statistic (Bar-Shalom et al., 2001; Chen et al., 2018):


$$\epsilon_{k}^{j}={\left({\text{e}}_{k}^{j}\right)}^{T}\,{\left(S\right)}^{-1}\,{\text{e}}_{k}^{j}$$

Here ${\text{e}}_{k}^{j}={\left[\begin{array}{ccc} ||{\text{e}}_{k,a}^{j}|| & ||{\text{e}}_{k,f}^{j}|| & ||{\text{e}}_{k,\omega }^{j}|| \end{array}\right]}^{T}$ is the innovation (i.e., sensory conflict) vector for hypothesis *j* and **S** is the scaled biological noise covariance (see Supplementary Table 1 for specific values of all parameters, and Kravets et al. (2021) for further discussion).

The NIS statistic informs the likelihoods of the incoming measurements, *y*_*k*_, given the *jth* gravity hypothesis, $x_{k}^{j}$, as follows:


$$p\,\left(y_{k}|x_{k}^{j}\right)=𝒩\,\left({\text{e}}_{k}^{j};0;S\right)=\frac{1}{\sqrt{{\left(2\,\pi \right)}^{n}\,\left|S\right|}}\,e^{-\frac{1}{2}\,\epsilon_{k}^{j}}$$

where $𝒩\,\left({\text{e}}_{k}^{j};0;S\right)$ is a Gaussian probability distribution function evaluated at ${\text{e}}_{k}^{j}$ and *n* is the length of ${\text{e}}_{k}^{j}$. The set of likelihoods is then used to calculate the Bayesian posterior probability of each gravity hypothesis:


$$p\,\left(x_{k}^{j}|Y_{1:k}\right)=\,\frac{p\,\left(y_{k}|x_{k}^{j}\right)\,p\,\left(x_{k}^{j}\right)}{p\,\left(y_{k}\right)}$$

where $p\,\left(x_{k}^{j}\right)$ is the prior probability density for the *jth* hypothesis of gravity at timestep *k* (the posterior of the previous time step), and *p* (*y*_*k*_) is the marginal likelihood that the measurement *y*_*k*_ was observed. Using the posterior probability densities of each gravity hypothesis in the current time step, a central estimate of the magnitude of gravity is calculated. A variety of summary statistics can be utilized for this calculation, but we present results based on the maximum *a posteriori* (MAP) estimator,


$${\hat{x}}_{\text{k},\text{MAP}}=a\,r\,g\,m\,a\,x\,\left[p\,\left(x_{k}|Y_{1:k}\right)\right]$$

We denote the estimate of the magnitude of gravity as $\left|\hat{g}\right|$. A “central observer” is then conditioned on the estimate, generating the overall model’s current estimate of linear acceleration, angular velocity, and the full gravity vector (i.e., tilt perception). The central observer represents an individual’s current model of self-orientation perception.

In Kravets et al. (2021), a static set of hypotheses for the magnitude of gravity were pre-defined and maintained. While this was sufficient to enable adaptation of the central estimate of the magnitude of gravity by Bayesian updates of the posterior probabilities, it fails to define a mechanism for the brain to produce, maintain, or remove potential alternative hypotheses. Here we employ a novel approach for how the set of alternative hypotheses (particles) for the magnitude of gravity evolve. In preparation for the next time step, a new set of hypotheses (for the magnitude of gravity) is sampled from a dynamic state transition probability density function. We propose that this state transition function is comprised of (1) a short-term memory, sensory conflict driven component and (2) a long-term memory component, which can be dynamically updated. This state transition probability is conveyed here in a general form and is fully defined in in the following sections:


$$x_{k+1}^{j}∼p\,\left(x_{k+1}^{j}|x_{1:k}^{j}\right)$$

Because we have set the particle sample distribution to be equivalent to the state transition probability, the recursive particle weight calculation (Ristic et al., 2003) becomes the following at each time step:


$${\tilde{w}}_{k}^{j}=p\,\left(y_{k}|x_{k}^{j}\right)\,w_{k-1}^{j}$$

After which, all particles are normalized so that cumulative probability is unity:


$$w_{k}^{j}=\frac{{\tilde{w}}_{k}^{j}}{\sum_{j=1}^{N_{s}}{\tilde{w}}_{k}^{j}}$$

### 2.2. Short-term memory search

**FIGURE 2:**
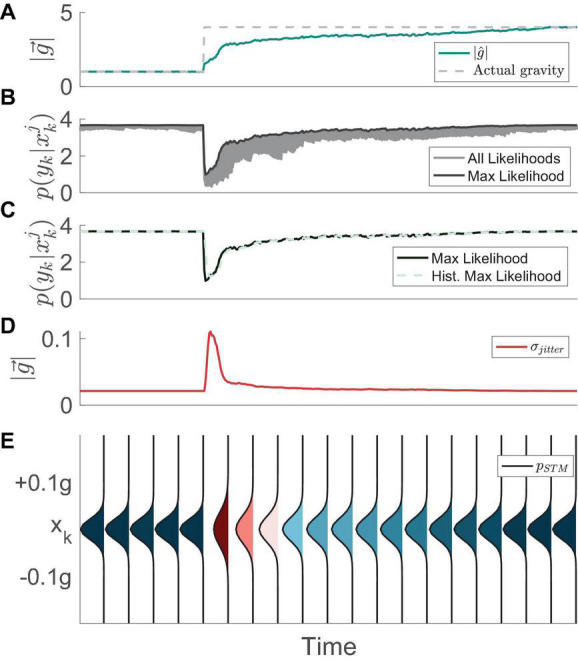
Example evolution of the STM mechanism over time, as proposed by Kravets et al. (2022). **(A)** The neural estimate of the magnitude of gravity (MAP) initially is identical to the actual magnitude of gravity (1 g). However, when the actual gravity instantly changes to 4 g in this simulation, the internal estimate gradually updates and converges to the proper value. **(B)** All particle computed likelihoods (gray) as well as the max computed likelihood (black). When the actual magnitude of gravity suddenly changes to 4 g, all of the particles produce low likelihoods. **(C)** The max likelihood (black) is used to compute a history of max likelihood (HML). **(D)** The evolution of the standard deviation of the STM function over time. **(E)** The STM probability density evolving over time. In instances where the history of the max likelihood drops the standard deviation of the probability density function increases. As we have done previously (Kravets et al., 2021), in this figure and throughout, the “Time” on the *x*-axis does not include units, as it depends on parameters in the model that can be tuned and fit to empirical data in the future.

Similar to the methods described in Kravets et al. (2022), we begin by recognizing that the CNS achieves adaptation to environmental stimuli, transitioning from a state of exploitation (i.e., using high probability hypotheses for the internal estimate of the magnitude of gravity) to a state of exploration (i.e., aggressively considering a wider range of alternative hypotheses), and eventually back to a state of exploitation. To mathematically capture this, we first define a “short-term memory” (STM) search capability of expanding and contracting the search domain in the presence of changing environmental stimuli (Figure 2). We define the STM search in general terms as the following Gaussian probability density function:


$$p_{S\,T\,M}\,\left(x_{k+1}|x_{k};\sigma_{J\,i\,t\,t\,e\,r}\right)=𝒩\,\left(x_{k},\sigma_{J\,i\,t\,t\,e\,r}\right)$$

Where *x*_*k*_ is the hypothesis of the magnitude of gravity at time step *k*. The short-term search is defined by a normal distribution with its mean chosen from the cumulative probability distribution of gravity hypotheses from the previous time step. The standard deviation of this normal distribution, σ_*Jitter*_, serves to provide “jitter” to the probabilistic sampling distribution. The value for σ_*Jitter*_ is defined to be inversely proportional to the maximum likelihood of the alternative hypotheses within a short-term history prior to the present time step, termed *HML*:


$$\sigma_{J\,i\,t\,t\,e\,r,k}\propto \frac{1}{{\left(H\,M\,L_{k}\right)}^{\chi_{1}}}$$

When calculating σ_*Jitter*_, we use the exponent χ_1_ to modulate the sensitivity of the algorithm to changes in maximum likelihood, such that a higher χ_1_ will lead to a more drastic increase in jitter when the maximum likelihood drops. The history of maximum likelihoods (HML) at time step *k* is calculated as an exponentially weighted average of the time history of maximum likelihoods:


$$H\,M\,L_{k}=\left(1-\frac{1}{1+f}\right)\,\left(H\,M\,L_{k-1}\right)+\frac{1}{1+f}\,m\,a\,x\,\left[p\,\left(y_{k}|x_{k}\right)\right]$$

Where *p*(*y*_*k*_|*x*_*k*_) is the set of likelihoods at time step *k* and *f* is the “forgetting factor,” which can range from 0 to 1. With this formula, the magnitude of the weighting factors on each historical maximum likelihood decreases exponentially as the age of the data increases. The forgetting factor determines the rate at which historical data is “forgotten,” with an *f* closer to 1 attributing more weight to past data. As the likelihoods are a function of sensory conflict, this averaging method prevents the model from becoming unstable with single unexpected measurements, and instead requires a buildup of sensory conflict before entering an “exploration” phase.

This formulation of jitter is not to be confused with the regularized particle filter (Ristic et al., 2003), and its original implementation comes from Kravets et al. (2022), utilized as a sample distribution. Functionally, the standard deviation of the short-term search increases when the history of the max likelihood decreases. Conversely, the standard deviation decreases when the likelihood function increases. This fluctuation represents a tradeoff between exploration and exploitation, respectively. Specifically, when the maximum likelihood of the alternative hypotheses for the magnitude of gravity has recently been high (i.e., the brain has been certain about the magnitude of gravity), it samples new hypotheses very near to the current ones (exploitation). An example of this process is provided in Figure 2.

### 2.3. Long-term memory search

In order to model CNS memory of learned sensorimotor states, we hypothesize that the CNS considers past, learned parameters comprising these states when formulating hypotheses. Regarding gravity transitions, we expect that an extended exposure to a certain gravity environment would eventually comprise a learned state of internal model parameters, and the CNS will consider these parameters when a substantial amount of sensory conflict (and by extension, substantial NIS) is present. A natural expression of this memory is through the state transition probability density function, similar to the STM search. Rather than just considering a localized (now called STM) search, as was the case in Kravets et al. (2022), a “long-term memory” search, *p*_*LTM*_ (*x*_*k* + 1_; *H*_*k*_), is now also considered.

Defining the long-term memory, *H*_*k*_ is the sequence of estimated states up to the current (*kth*) iteration that are *harmonious*. We definite the set of harmonious state estimates affecting long-term memory as those which produce a resultant NIS (i.e., central NIS estimate) beneath a threshold, ν :


$$H_{k}=\left\{h_{i},i=0,\ldots ,k\right\}$$

where *h*_*i*_ satisfy:


$$\epsilon_{i}\,\left(h_{i}\right)<\nu$$

Enabling the dynamic evolution of learned states, we model the long-term portion of the state transition probability as a function of the time spent in harmonious states. The continuous time representation is the following:


$$p_{L\,T\,M}\,\left(x_{k+1};H_{k}\right)=\frac{1}{T_{w\,i\,n\,d}}\,\int_{t-T_{w\,i\,n\,d}}^{t}\delta \,\left(x_{k+1}-h\,\left(\tau \right)\right)\,d\,\tau$$

Here, *T*_*wind*_ is the temporal window of which the CNS considers past harmonious states (presumably only considering a finite amount of information). In discrete time form, considering a discrete set of harmonious states, this evolution becomes the following:


$$p_{L\,T\,M}\,\left(x_{k+1};H_{k}\right)=\frac{1}{N_{w\,i\,n\,d}}\,\sum_{i=k-N_{w\,i\,n\,d}}^{k}\delta \,\left(x_{k+1}-h_{i}\right)$$

$$N_{w\,i\,n\,d}=\frac{T_{w\,i\,n\,d}}{\Delta_{k}}$$

**FIGURE 3:**
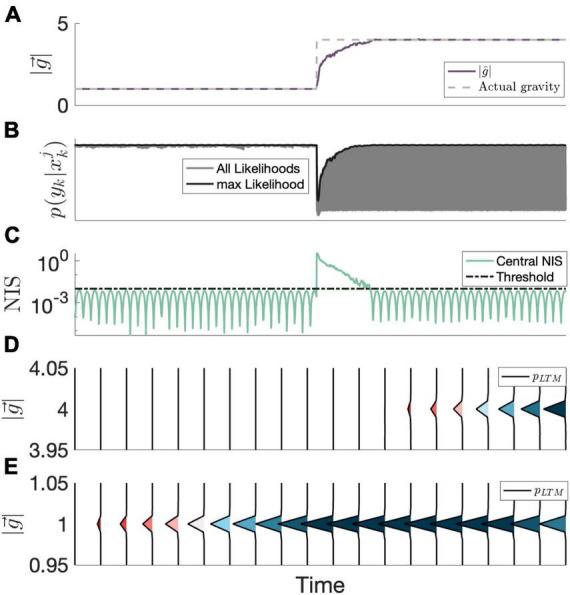
Example evolution of the proposed LTM mechanism over time. **(A)** The neural estimate of the magnitude of gravity (MAP) is shown, along-side the actual magnitude of gravity. **(B)** All particle likelihoods (gray) as well as the max likelihood (black). In contrast to the likelihoods at 1 g, the likelihoods following the transition to 4 g span a wider range, as the CNS considers a learned state at 1 g. **(C)** The central observer’s NIS statistic, which determines whether or not the current estimate is harmonious (i.e., beneath an internal threshold; dashed line). **(D)** The LTM probability density evolving over time around 4 g. **(E)** The LTM probability density evolving over time around 1 g. Note that the LTM probability density between 1.05 and 3.95 is negligible (i.e., is 0) and not shown, so between panels **(D,E)**, the entirety of the LTM probability density function is shown. In instances where the current estimate is harmonious, the long-term memory portion of the state transition probability density function (shown here evolving over time) is updated, storing the state information at the current estimate. Probability densities are colored by peak densities to demonstrate emergence and cessation of learned states.

For the long-term (and short-term) memory, there is an implicit reliance on past parameters (*x*_1:*k*_ = {*x*_1_, …*x*_*k*_}) and measurements (*Y*_*1:k*_) since *H*_*k*_ (and σ_*Jitter*_) are dependent on both of these sets. With this mathematical framework, we can model the evolution of learned states considered by the CNS when evolving hypotheses without making prior assumptions about what the learned states are, and these learned states construct themselves from states with sub-threshold sensory conflict without access to ground truth information. An example of this process is provided in Figure 3.

### 2.4. The state transition probability density function

We represent the full state transition probability as a combination of both short-term and long-term conditional state transition probabilities, expressed as the following mixture model:


$$p\,\left(x_{k+1}|x_{k};\sigma_{J\,i\,t\,t\,e\,r},H_{k},W\right)=$$

$$\frac{p_{S\,T\,M}\,\left(x_{k+1}|x_{k};\sigma_{J\,i\,t\,t\,e\,r}\right)*\left(1-W\right)+p_{L\,T\,M}\,\left(x_{k+1};H_{k}\right)*W}{\int_{-\infty }^{\infty }\left[p_{S\,T\,M}\,\left(x_{k+1}|x_{k};\sigma_{J\,i\,t\,t\,e\,r}\right)*\left(1-W\right)+p_{L\,T\,M}\,\left(x_{k+1};H_{k}\right)*W\right]\,d\,x}$$

**FIGURE 4:**
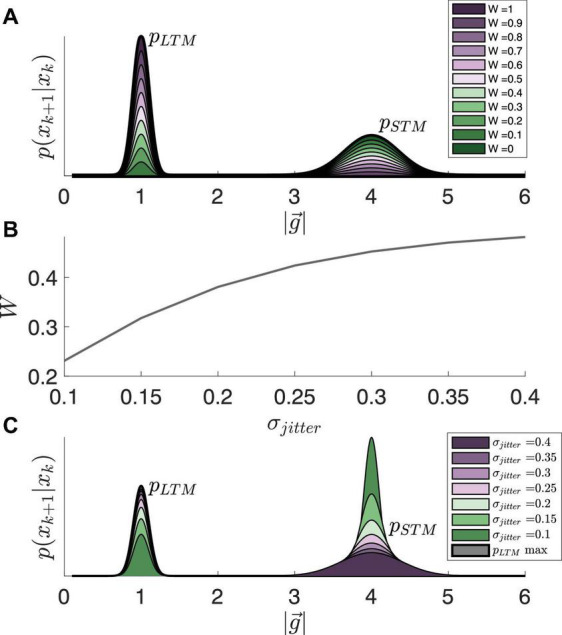
**(A)** Impact of weighting parameter, ***W***, on prioritization of long-term over short-term memory. **(B)** Modeling ***W*** as the solution to a logistic function, dependent on **σ**_***Jitter***_. **(C)** The resultant state transition probabilities due to varying levels of jitter, displayed in panel **(B)**.

Here, *W* is a parameter that defines the prioritization of long-term over short-term memory (the impact of this parameter is conveyed in Figure 4A). We choose to model this weighting parameter as a function of the jitter (and as a result, related to both likelihood and sensory conflict) via a sigmoid function, lending weight to STM when in an exploitative state and more equally weighting both STM and LTM while in an exploratory state:


$$W_{k}=\frac{1}{1+e^{-\sigma_{j\,i\,t\,t\,e\,r}\cdot \chi_{2}}}-\frac{1}{2}$$

and now, *W*$\in \left(0,\frac{1}{2}\right)$. How the full state transition probability function varies based on this parameter is conveyed in Figures 4B, C.

## 3. Example model simulations

### 3.1. Model implementation

The theoretical framework described above is implemented herein both with and without LTM, using the MAP estimate for the estimate of the magnitude of gravity ($\left|\hat{\text{g}}\right|$), in order to demonstrate the effect of modeling memory on various adaptation profiles. The central observer includes the recent enhancement to the observer model that incorporates differential weighting of components of otolith stimulation (Clark et al., 2015c), allowing for prediction of roll tilt over- or underestimation in hyper- and hypo- gravity scenarios. As was the case in prior models (Kravets et al., 2021), units of time on the *x*-axis are excluded until free parameters can be determined through future controlled experiments. However, all model parameters are held constant across model simulations to enable comparisons between adaptation profiles.

The motion profile influencing adaptation in each of the example simulations is a passive sinusoidal roll tilt at 1 rad/s (0.159 Hz) angular frequency with a peak angular velocity of 10°C. Because the observer model is implemented in Simulink, full re-run of the Simulink model from the beginning of the simulation time is required every time a particle’s estimate of gravity changes (i.e., at every time step). To circumvent the computational expense associated with this process, all potential gravity levels (on a grid with a coarseness of 1/100 g) and their associated sensory conflicts were pre-computed to be pulled from a bank during the particle filter simulations. To reduce the variability in the simulations for the purpose of comparison, we chose to use *N*_*s*_ = 100 particles within the particle filter (but adaptation can be achieved with only a few particles). Additionally, similar to the design considerations of a Sequential Importance Resampling (e.g., “Bootstrap”) Particle Filter (Ristic et al., 2003), we chose to resample particles from the posterior distribution at every iteration. This reduces the recursive particle weight calculation to be proportional to their likelihoods.

We also introduced sensory noise into the vestibular sensory signals similarly to Kravets et al. (2021). Currently, the exact form of actual sensory vestibular noise is unknown. Multiple works have theorized that increased vestibular stimuli, particularly to the otolith organs in the presence of hyper-gravity, results in an increased signal to noise ratio (and decreased ratio in hypo-gravity) (Clark et al., 2015a; Rosenberg et al., 2018). Other works suggest that vestibular sensory noise increases non-linearly with vestibular stimuli (Vingerhoets et al., 2009). We implemented vestibular noise similarly to prior works (Kravets et al., 2021, 2022) where a constant noise power (1e-8) is applied to the vestibular sensory signals (noise power is the height of the power spectral density of the white noise added to the system). A comparison of noise power to adaptation time can be found in Kravets et al. (2021; see Figure 7).

### 3.2. Simulation results

**FIGURE 5:**
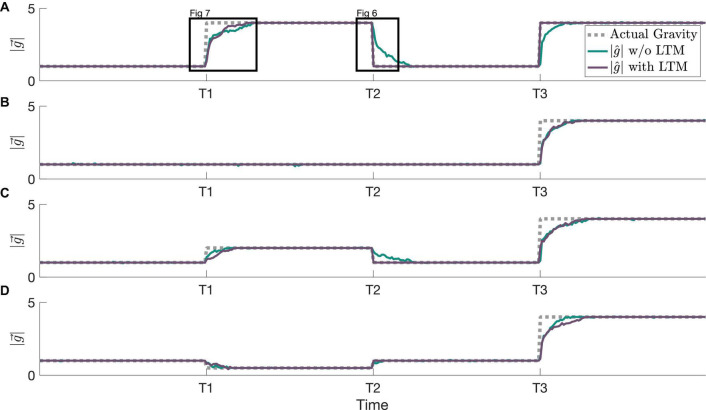
Example simulations showing various adaptation profiles and resulting MAP estimates, both with and without the LTM component of the state transition probability. All simulations begin with an actual gravity level of 1 Earth gravity, and all *x*-axes are linked to the same timescale. **(A)** An adaptation to 4 g (see Figure 7), readaptation to 1 g (see Figure 6), and readaptation to 4 g. **(B)** An adaptation to 4 g after a prolonged stint in 1 g. **(C)** An adaptation to 2 g, readaptation to 1 g, and an adaptation to 4 g (a second novel hyper-gravity stimulus). **(D)** An adaptation to 0.5 g, readaptation to 1 g, and an adaptation to 4 g (a second novel gravity stimulus, but where the first was hypo-gravity, and the second is hyper-gravity).

In Figure 5, our simulations demonstrate how this framework, incorporating LTM, is able to achieve faster readaptation to familiar stimuli than the prior framework with only the jitter (implemented as STM here) component from Kravets et al. (2022). The relative performance of these two frameworks is compared across multiple adaptation scenarios. At timepoint T2, readaptation to 1 g differs when implementing LTM due to the learned 1 g state prior to timepoint T1. In Figure 5A, readaptation to 4 g at T3 occurs at an accelerated rate due to the learned 4 g state between T1 and T2. The formation of the internal magnitude of gravity estimate is accelerated at T3 only in Figure 5A, and prior gravity transitions to other magnitudes of gravity (staying at 1 g, transitioning to 2 g, and transitioning to 0.5 g in Figures 5B–D, respectively) do not affect the adaptation rate to 4 g.

**FIGURE 6:**
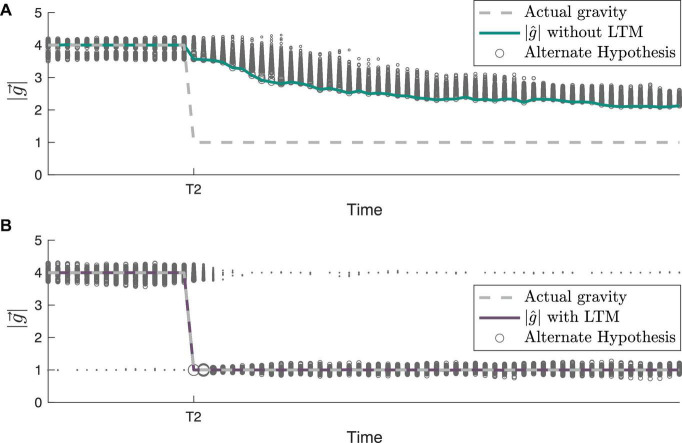
Gravity hypothesis generation with and without long-term memory (LTM). The path of gravity adaptation differs between simulations **(A)** without LTM incorporated and **(B)** with LTM incorporated, as shown by the small particles/alternate hypotheses at 1 g prior to T2 (see Figure 5A for full gravity transition history). In both panels **(A,B)**, the sizes of the particles are proportional to the posterior probability of each hypothesis at that timestep.

To further demonstrate the inner workings of our framework incorporating LTM, we examine the evolution of individual hypotheses (particles) considered by the CNS over time. Both their dynamic evolution and the posterior weighting of these hypotheses are compared with and without LTM in Figure 6, an enhanced view of timepoint T2 from Figure 5A. Without LTM (Figure 6A), just prior to timepoint T2, the gravity hypothesis particles are exclusively focused around the actual gravity level of 4 g. However, with LTM (Figure 6B), because 1 g is a previously learned state, a few of the gravity hypothesis particles continue to be sampled at 1 g. This enables a very different time course of adaptation of the internal magnitude of gravity. Without LTM, the RBPF has to gradually resample the hypotheses. Those gravity hypotheses that are lower magnitude (closer to 1 g) produce higher likelihoods, which encourages subsequent sampling of lower gravity magnitudes, but it is still a gradual process. In contrast, with LTM (Figure 6B), the internally estimated gravity level corrects to 1 g very rapidly after the actual gravity magnitude transitions from 4 to 1 g at timepoint T2. This is because gravity hypothesis particles are already being intermittently sampled at 1 g (as a learned state), such that when the actual gravity changes to 1 g the likelihood of the particle near 1 g is very high. It takes a little time after T2 for the majority of the particles near 4 g to transition to being centered around 1 g, but the central estimate of the gravity magnitude converges nearly immediately. Further, with LTM, the learned state of 4 g continues to be intermittently sampled by the gravity hypothesis particles after T2.

**FIGURE 7:**
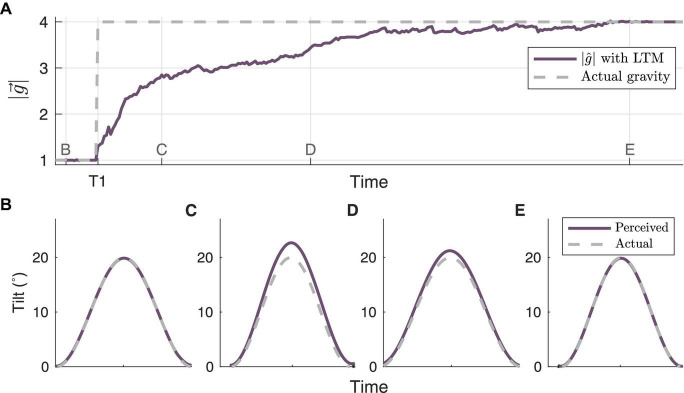
Model simulation of tilt perceptions associated with adaptation to gravity transition. **(A)** highlights the adaptation trajectory surrounding the gravity transition from 1 to 4 g at timepoint T1 in Figure 5, with a roll tilt motion profile. The associated tilt perceptions (or misperceptions) generated by the central observer at time points **(B–E)** are shown in their respective panels.

Finally, we simulate human perception of roll tilt (Figure 7) during the central adaptation occurring at T1 in Figure 5A. This simulation was conducted to demonstrate how the framework presented enables a better understanding of transitory perceptions during central adaptation, much like in Kravets et al. (2021). At timepoint B, when $\left|\hat{\text{g}}\right|$ correctly matches the actual gravity (prior to the gravity transition at T1), the perceived tilt accurately matches actual tilt (i.e., no misperception). However, following the gravity transition at T1, and before the model has time to fully adapt to the new gravity level, the model predicts an overestimation of tilt, which is evident at timepoint C. This is consistent with the “G-Excess” illusion, in which upon initial exposure to hyper-gravity, humans misperceive self-tilt as being larger than it actually is (Schöne, 1964; Correia et al., 1968; Clark et al., 2015b). The misperception decreases by timepoint D, as $\left|\hat{\text{g}}\right|$ more closely matches the actual gravity level. By timepoint E, the model has correctly learned the new gravity level, and once again the predicted perception matches the actual tilt.

## 4. Discussion

### 4.1. Summary of theoretical contributions

The overarching computational model provided herein of vestibular adaptation to gravity transitions enables modeling transient states of sensorimotor impairment due to changing gravitational stimuli. Our framework is dependent on vestibular sensory stimuli alone, without access to other channels of sensory information or direct information about the true magnitude of gravity. This model CNS recursively estimates gravity’s magnitude through Bayesian inference, as previously proposed in Kravets et al. (2021). However we differ from the Kravets et al. (2021) implementation (which considered a static set of gravity hypotheses at each time step), and instead utilize methods proposed in Kravets et al. (2022) by modeling the evolution of potential hypotheses via an indirect sampling approach, characterized by a Rao-Blackwellized particle filter algorithm.

As a novel improvement to these previous implementations, the model framework presented here emulates sensorimotor adaptation while retaining a memory of past harmonious states, allowing the model to consider previously experienced gravity levels (while also dynamically learning new states) when formulating new parallel alternative hypotheses of gravity. This framework’s contributions include both modeling the influence of learned internal state parameters on the CNS’s evolution of hypotheses and a model of the temporal evolution of learned internal state parameters (corresponding to implicit memory). Together, this framework enables the consolidation of harmonious states into long-term memory, in turn enabling faster readaptation. Notably, the computations involved with learning and storing internal state parameters do so without any ground-truth knowledge of the actual magnitude of gravity at any point in time. Further, this long-term memory functionality is beneficial toward readapting to a previously experienced magnitude of gravity, but does not have any substantial downside (i.e., it helps when useful, but does not hurt when the learned state is not relevant). While we propose this framework specifically for adaptation to a changing gravitational stimulus by learning magnitudes of gravity, this framework can be applied more broadly to other model parameters (e.g., other observer model gains and / or time constants) and other forms of implicit memory (e.g., consolidating descriptive parameters within internal models describing motor control).

### 4.2. Insight from model simulations

Compared to modeling STM alone, the LTM framework enables exploitation of information from past learned states to achieve faster readaptation. This effect is best demonstrated in Figure 5A at T2, when readaptation to 4 g from 1 g occurs. When considering new hypotheses of gravity, the STM search is restricted to a domain (represented here by a Gaussian distribution) centered around the current hypotheses. However, the LTM framework retains a finite memory of previously experienced harmonious states to additionally consider when in the CNS is in a state of exploration. This effect is demonstrated in Figure 6, readaptation to 1 g from 4 g. The rate of adaptation to learned states can be modulated through the parameter *W* and additionally through the free parameter χ_*2*_. If *W* is sufficiently low, the adaptation trajectories with and without LTM are indistinguishable. Further, because LTM is modeled with a finite retention window, learned states are down-weighted (i.e., reduced probability of being considered by the CNS), taking longer for readaptation occur, and eventually unlearned entirely if enough time is spent outside the learned state.

Despite the more rapid readaptation enabled by the LTM framework when memory of a harmonious state exists, the time course of adaptation to novel gravity environments remains the same both with and without LTM in the simulations shown. This is consistent with evidence that sensorimotor learning is restricted to specific conditions (Tjernström et al., 2002). However, the LTM framework has the potential to facilitate quicker adaptation to unfamiliar gravity conditions that closely approximate a previously learned gravity environment. For example, if the model has a history of adaptation to 3 g, it may adapt more rapidly when transitioning from 1 to 3.2 g because it will start “exploring” new particles surrounding 3 g (which will have higher likelihoods that the existing particles at 1 g) instantly. While this hypothesis would need to be tested experimentally, it highlights one of the benefits of a computational model of gravity adaptation, as it provides specific quantitative theories that can inspire experimental work.

Finally, this framework provides the utility of computationally generating self-orientation perceptions in humans during the period of time where adaptation occurs, dependent on previous exposure to gravitational environments. It has previously been shown that tilt is overestimated following a transition to hyper-gravity from 1 g before adaptation is fully achieved (Clark et al., 2015b). Similar to Kravets et al. (2021), the internal estimate of gravity driven by this framework results in an overestimation of tilt during adaptation to a greater magnitude of gravity (shown in Figure 7), and the LTM framework presented herein enables computational assessments of self-orientation perception that are affected by memory of past states. By using this model’s predicted perceptions in response to a controlled physical stimulus, the results can be mapped to experimental results and operational concerns. In the current model implementation, the central observer produces predictions of spatial orientation perception using the central estimate of the magnitude of gravity. One alternative approach would be to have each parallel, alternative observer predict spatial orientation perceptions, which could then be weighted and merged. This approach would allow for the quantification of bimodal orientation perceptions as have been reported for some motion paradigms (Vingerhoets et al., 2008).

### 4.3. Limitations and future work

Once again, this model exists as an untuned and unvalidated theory. As a result, the time course of adaptation remains undetermined. While relative comparisons can be made between simulations, there exists a need to obtain empirical data describing the time course of adaptation. For transitions from 1 g to microgravity, recent work has provided some (while limited) insight. In an attempt to quantify in-flight adaptive changes, performance in a bimanual coordination task in-flight was not found to be correlated to mission duration exceeding 4 months (spanning 4 to 11 months) (Tays et al., 2021). This finding reinforces the concept that sensorimotor adaptation reaches an exploitation state following an exploration state once the CNS achieves some desirable level of adaptation. Additionally, this finding of no differences after 4 months may provide some upper-bound time course of functional adaptation to microgravity. However, future work measuring perceptions during adaptation is recommended to quantify the time course of adaptation.

The simulation results presented here focus on gravity transitions between 1 g, hyper-gravity, and hypo-gravity, and intentionally do not address a transition to or from microgravity (i.e., 0 g). Modeling adaptation to microgravity is a unique challenge that likely involves more than just a reinterpretation of the internal magnitude of gravity. Evidence suggests that the CNS may reinterpret all otolith stimulation as translation (Young et al., 1984; Parker et al., 1985), or that there may be a degradation of the internal model of how rotational cues affect tilt perception (Merfeld, 2003). Notably when utilizing this framework for a transition to microgravity, as the internal estimate of gravity approaches zero, the central observer begins to interpret otolith stimulation as linear acceleration, and upon reaching zero, all otolith stimulation is interpreted as linear acceleration. When transitioning back to a 1 g environment, when the estimate of gravity ∈ (0, 1*g*), the central observer predicts linear acceleration perceptions opposite that of physical tilt, similar to those predicted by the rotation otolith tilt-translation reinterpretation hypothesis. Despite these promising effects, it is possible that additional model parameters associated with the GIF [such as the *K*_*f*_ and *K*_*f*ω_ gains in the observer model, see Clark et al., 2019 for details] also change. Alternatively, the observer framework may fundamentally change upon transitioning to microgravity in a way that cannot be reflected through updating values of model parameters, and it is possible that these changes differ between individuals. While the implementation of our model does not address these unique challenges associated with microgravity, this modeling framework is not limited to just a dynamic adjustment of the magnitude of gravity and could be used to include adaptation of additional parameters that may be involved in transitions to microgravity. Future works should thoroughly explore the extension of this framework to microgravity.

It is also important to emphasize that the implementation of the model we have presented is limited to vestibular cues resulting from passive motion. However, when undergoing a gravity transition, the CNS is likely to use all sources of sensory information, such as visual and somatosensory cues, to adapt to the new environment. In fact, there is evidence that the CNS may reweight the sensory information it receives based on the reliability of the cues it is receiving (Fetsch et al., 2009; Hupfeld et al., 2022). While this is a limitation of the current implementation of the proposed framework, the model could be adjusted to include these cues and preferential weightings between sensory systems. Versions of the observer model have been developed to include visual cues (Newman, 2009; Clark et al., 2019), and other adjustments to the sensory dynamics process could be incorporated.

Sensory reweighting can be modeled by modulating the gains in the observer model contributing to perception from different sensory channels [e.g., the visual channel *K*_*v*_*v*__ and *K*_ω_*v*__ gains in the visual observer model, see both (Newman, 2009; Clark et al., 2019) for details]. Similar to our demonstration of updates to the magnitude of gravity parameter over time, these parameters may be updated through indirect sampling and Bayesian inference, driven by sensory conflict. Our LTM framework can also be leveraged for modeling learned states comprising different sensory weighting schemes over time. Moreover, the sensory conflict from this additional sensory channel can be incorporated into the NIS statistic formulation. To garner a full picture of sensory adaptation, the addition of somatosensory pathways and variable weighting should also be modeled. Providing more reliable sensory cues may affect the rate of adaptation predicted by the model.

As theories and evidence surrounding specific adaptation scenarios and multisensory integration mature, our proposed modeling implementation can be modified accordingly. Building up the framework presented here (through empirical validation, multisensory modeling, and reweighting of sensory channels) may eventually result in a means of improving training, operational scheduling, and countermeasure development accompanying planned gravity transitions. This framework represents a foundational stepping stone toward these goals.

## Data availability statement

The original contributions presented in the study are included in the article/Supplementary material, further inquiries can be directed to the corresponding author.

## Author contributions

AA, VK, and TC contributed to the theoretical framework and wrote sections of the manuscript. AA, VK, TC, and NA contributed to the functionality of the computational model and the reviewing of simulation results. AA and VK implemented the model. All authors contributed to manuscript revision, read, and approved the submitted version.
