# Supplementary material for: Modeling orientation perception adaptation to altered gravity environments with memory of past sensorimotor states
Source: Front Neural Circuits. 2023 Jul 20;17:1190582. doi: 10.3389/fncir.2023.1190582 (PMC10399228; doi:10.3389/fncir.2023.1190582)
Supplement: Supplementary file 1 [file Table_1.docx]

Supplementary Material

Modeling Orientation Perception Adaptation to Altered Gravity Environments with Memory of Past Sensorimotor States

Aaron R. Allred^1†^, Victoria Kravets^1†^, Nisar Ahmed^2^, Torin K. Clark^1*^

^1^Bioastronautics Laboratory, Smead Department of Aerospace Engineering Sciences, University of Colorado – Boulder, Boulder, CO, United States

^2^COHRINT Laboratory, Smead Department of Aerospace Engineering Sciences, University of Colorado – Boulder, Boulder, CO, United States

*** Correspondence:**
**Torin K. Clark**
[**torin.clark@colorado.edu**](mailto:email@uni.edu)

†These authors contributed equally to this work and share first authorship

**Supplementary Table 1.** Model Parameters. To initialize the model, *Ns* number of particles are sampled from a normal distribution. The biological noise covariance matrix, weighted by *Ks*, is used to calculate the NIS statistic and likelihood of the gravity hypotheses at each time step. The forgetting factor determines the rate at which the historical data is ‘forgotten’ when calculating the history of max likelihoods. The ‘harmonious’ threshold determines when gravity values are added to the LTM distribution. The base jitter multiplier scales the jitter calculation at each time step, and the jitter power controls the sensitivity of the jitter calculation to changes in maximum likelihoods. $\chi_{2}$ is a free parameter in the calculation of *W* (i.e., the dynamic weight given to the LTM).

| Parameter | Value |
| --- | --- |
| Number of Particles, *Ns* | 100 [unitless] |
| Particle distribution initialization | $N(\mu= 1, \sigma= 1x{10}^{-2})$ |
| Biological noise covariance matrix, *S* | $\left[ \begin{matrix} {\sigma_{a}}^{2} & 0 & 0 \\ 0 & {\sigma_{f}}^{2} & 0 \\ 0 & 0 & {\sigma_{\omega}}^{2} \end{matrix} \right]$ ,where $\sigma_{a}= 0.0024$[g’s]*, $\sigma_{f}=0.0024$[rad] and $\sigma_{\omega}=0.003$ [rad/s] |
| Noise covariance weighting parameter, *Ks* | $1x{10}^{4}$ [unitless] |
| Forgetting factor in exponential weighting equation, *f* | 1 [unitless] |
| ‘Harmonious’ memory threshold, $\boldsymbol{\nu}$ | 0.01 [unitless] |
| Jitter power, $\boldsymbol{\chi}_{\boldsymbol{1}}$ | 1.5 [unitless] |
| Base jitter multiplier | 0.15 [g’s] |
| Dynamic *W* free parameter, $\boldsymbol{\chi}_{\boldsymbol{2}}$ | 10 [unitless] |
| *W* initialization | 0.075 [unitless] |

* Karmali, F., and Merfeld, D. M. (2012). A distributed, dynamic, parallel computational model: the role of noise in velocity storage. J. Neurophysiol. 108, 390–405. doi: 10.1152/jn.00883.2011
